# Dynamic Trends in Aquatic Product Supply and Consumption in China: Implications for Sustainable Diets and Environmental Impact Reduction

**DOI:** 10.3390/foods14020191

**Published:** 2025-01-09

**Authors:** Wanni Yang

**Affiliations:** Research Institute for Eco-civilization, Chinese Academy of Social Sciences, No. 27 Wangfujing Street, Dongcheng District, Beijing 100710, China; wanniyang@pku.edu.cn

**Keywords:** aquatic products, food system transformation, carbon footprints, water footprints, food security, marine fisheries

## Abstract

Aquatic foods play a pivotal role in transforming food systems. As the world’s leading producer, consumer, and trader of aquatic products, China’s potential for sustainable supply and consumption is critical to understand. The aim of this study was to depict the dynamic trends of aquatic products and the consequences of sustainable diets and environmental impacts. A panel dataset about Chinese aquatic products covering the period from 1952 to 2023 was drawn for analysis. Diet sustainability was assessed with the deviation from the Dietary Guidelines for Chinese Residents (2022) and EAT-Lancet recommendations. The environmental impacts of aquatic products’ supply and consumption were assessed using carbon footprints and water footprints. The findings reveal that aquatic products’ supply increased from 4.65 million tons to 71.16 million tons from 1978 to 2023, and annual aquatic food consumption per capita increased from 3.50 kg in 1978 to 15.20 kg. While overall supply meets consumption needs, structural imbalances persist at the provincial level. Over time, the influence of marine fishery products has declined from 1.06 million tons (63.63%) in 1952 to 35.85 million tons (50.38%) in 2023, whereas offshore aquaculture shows promising potential for meeting future supply demands (23.96 million tons in 2023, accounting for 66.82% of marine fishery production). To align with healthy dietary goals and environmentally sustainable food systems, provincial aquatic food demand across China was adjusted. The carbon footprints and water footprints of both current and adjusted consumption patterns were also assessed. The results indicate that adjusting consumption based on the Dietary Guidelines for Chinese Residents (2022) and EAT-Lancet recommendations could reduce environmental impacts to different degrees. The findings could offer valuable references and insights into developing sustainable strategies in aquatic product management and advancing food system transformation.

## 1. Introduction

As the world’s largest producer, consumer, and trader of aquatic products [1], China plays a central role in the global food system and marine resource management [2]. With its vast and diverse aquatic ecosystems, China’s fisheries contribute significantly to global food security, providing essential nutrients to billions of people. However, the rapid expansion of China’s aquatic product supply and consumption has led to concerns about the long-term sustainability of its fishing practices and their environmental impacts [1,3,4,5]. As China’s aquatic food sector continues to grow, understanding the dynamic characteristics of supply and consumption patterns, along with their ecological consequences, has become increasingly important for both national and global sustainability efforts [6,7].

Previous studies about aquatic products mainly focused on the fishery sector’s development itself. The studies about the global trade of aquatic products mainly focus on the economic impact of seafood trade on national and international markets [8]. Topics include trade policies, supply chains, market demand, and the economic implications of fisheries management practices [9,10]. The studies about aquaculture and technological innovation mainly focus on improving production efficiency, reducing environmental impact, and enhancing the health and sustainability of farmed species [11,12,13,14]. The studies about the environment and ecology usually focus on the effects of fishery practices on marine ecosystems [15,16,17]. Studies analyze the ecological consequences of unsustainable fishing, such as species depletion, disrupted food webs, and the degradation of aquatic habitats like coral reefs and wetlands [16,18,19]. Understanding how different fishing techniques and aquaculture methods affect marine biodiversity is crucial for developing policies aimed at minimizing environmental harm [20,21]. Another significant portion of recent research is dedicated to understanding the sustainability of aquatic product production and consumption. Scholars explore both the environmental impacts of fishing practices, such as overfishing, habitat destruction, and biodiversity loss, and the potential of aquaculture as a more sustainable alternative [11,12,16,18,19,22]. Research on the carbon and water footprints of aquatic food systems has also gained prominence as global concerns about climate change and resource depletion increase [20,23,24,25,26]. In recent years, more and more scientists have started to notice the role of aquatic products in global food security when discussing food system transformation [27,28,29,30]. This concern is even more pronounced in developing countries [31], where fisheries are crucial for nutrition and livelihoods. The impact factors that lead to aquatic food consumption transformation include economic growth, urbanization, and dietary preferences, impacting the availability and sustainability of aquatic foods and so on [32,33,34].

However, there is still a lack of studies on aquatic product supply and consumption considering both health effects and environmental benefits. Examining the shift toward more sustainable and healthier consumption practices requires efforts to improve diets through better management of aquatic food resources. This study aims to address these pressing challenges by analyzing the dynamic changes in China’s aquatic product supply and consumption. Specifically, the research questions of this study mainly include: (1) What are the dynamic trends in the supply and consumption of aquatic products in China during the past few decades? (2) What are the environmental impacts (carbon and water footprints) of current aquatic product consumption patterns in China? (3) How can shifting consumption patterns toward more sustainable practices, as recommended by the Dietary Guidelines for Chinese Residents (2022) and EAT-Lancet recommendations, reduce the environmental impacts of aquatic food consumption in China? (4) What role does aquaculture play in the sustainable supply of aquatic products in China, and how does it compare to traditional marine fisheries in terms of environmental sustainability? This study tried to draw on a panel dataset about Chinese aquatic products from 1952 to 2023 for analyses to answer these research questions. The findings of this study could provide actionable insights for the policymakers into how China can shift towards a more sustainable and health-conscious food system.

## 2. Materials and Methods

### 2.1. Data Collection

This study mainly used various types of data, including statistical data on aquatic production supply, aquatic production trade, and aquatic food consumption, spatial data, data on food nutrition, and socioeconomic data (i.e., demographic data, etc.). These data cover from 1952–2023 and include both national and provincial level data.

The aquatic production supply and consumption data (1952–2023) were collated according to the data disclosed in *China Agricultural Statistics 1949–2019*, *China Fishery Statistics Yearbook* (2006–2024) and *China Fishery Statistics Bulletin 2023* [35], all of which were compiled from published yearbooks obtained from the National Library of China and official website of Ministry of Agriculture and Rural Affairs of the People’s Republic of China (https://www.moa.gov.cn/, accessed on 1 November 2024). Then, the aquatic production information, which was derived from the *China Rural Statistical Yearbook* (1988–2023) and *China Marine Economic Statistical Bulletin* (2018–2023), was used as supplementary sources and cross-validation for the above indicators.

Spatial data, which mainly includes country and provincial boundary data, were obtained from the Data Center for Resources and Environmental Sciences, the Chinese Academy of Sciences (CAS) (http://www.resdc.cn/, accessed on 1 November 2024). Food nutrition data about aquatic food and the ideal standards (Chinese Food Guild Pagoda 2022 and the planetary health diet) were obtained from (a) Dietary Guidelines for Chinese Residents (2022), released by the Chinese Nutrition Society [36]; (b) EAT-The Lancet Commissions [3]. Socioeconomic data were obtained from the official website of China National Bureau of Statistics (https://www.stats.gov.cn/, accessed on 1 November 2024).

### 2.2. Data Analysis Methods

#### 2.2.1. Conversion Method of Carbon Footprint and Water Footprint

Regarding the environmental footprint of aquatic products, this study mainly focused on the carbon footprint and water footprint of aquatic food consumption in this study.

Carbon footprint is a popular indicator to evaluate the impact of human food consumption activities on the environment [37]. Carbon footprint data for food from a lifecycle analyses (LCA) perspective are unavailable in China [38].·This study applied the carbon footprint coefficient of aquatic food as 3.85 kg CO_2-eq_/kg under the previous LCA framework [38]. The equation to calculate the carbon footprint of aquatic food consumption per capita is as follows:

(1) $${CF}_{i}=ACF\times Q_{i}$$

where *CF* represents the carbon footprint of aquatic food consumption per capita of province *i*, and the unit is kg CO_2-eq_; *ACF* represents the carbon footprint coefficient of aquatic food, and the unit is kg CO_2-eq_/kg; *Q_i_* represents the annual per capita consumption of aquatic products for province *i*, and the unit is kg.

Water footprint is another indicator used to evaluate the environmental consequences of food consumption activities. The water footprint refers to the cumulative virtual water content required for all products and services in a certain area, which mainly includes blue water footprint, green water footprint, and grey water footprint [39]. In this study, the author followed the water footprint coefficient of aquatic food as 1.22 m^3^/kg applied by Xu et al. [40]. The equation to calculate the water footprint of aquatic food consumption per capita is as follows:

(2) $${WF}_{i}=AWF\times Q_{i}$$

where *WF* represents the water footprint of aquatic food consumption per capita of province *i*, and the unit is m^3^; *AWF* represents the water footprint coefficient of aquatic food, and the unit is m^3^/kg; *Q_i_* represents the annual per capita consumption of aquatic products for province *i*, and the unit is kg.

#### 2.2.2. Statistical Analytical Strategy

**Figure 1:**
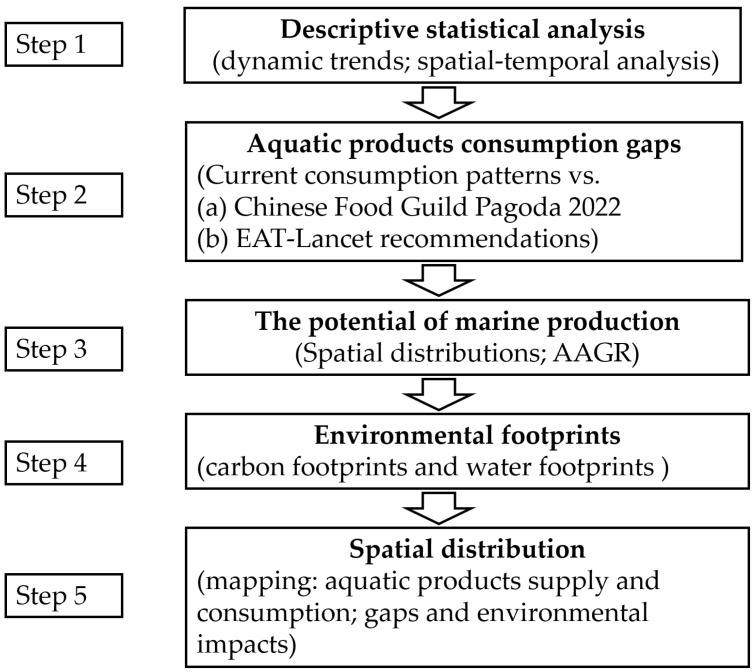
Data analysis procedure in this study. Note: abbreviation: AAGR—average annual growth rate.

This study’s analyses were conducted in five parts (Figure 1). First, time and spatial series descriptive statistical analysis was conducted, which aimed at identifying the aquatic production supply, the trend and potential of fishery (including aquaculture and capture fishery, marine fishery, and freshwater fishery), and the aquatic food consumption.

Second, this study analyzed the gaps between the annual per capita consumption of aquatic products among Chinese residents and (a) the Chinese Food Guild Pagoda 2022, and (b) EAT-Lancet recommendations. Additionally, the dynamic changes and trends of these gaps among different provinces were analyzed, revealing the potential of aquatic food consumption and supply in China as part of the country’s food system transformation.

Third, the potential of marine production was analyzed. Marine food product supply in twelve coastal provinces of China was analyzed. In addition, the average annual growth rate (AAGR) of marine product supply was calculated. The equation to calculate the AAGR is as follows:

(3) $$R=\left(\sqrt[{n-1}]{S_{n}/S_{1}}-1\right)\times 100\%\;$$

where *R* represents the average annual growth rate, *S* represents marine product supply, and *n* represents the time period.

Fourth, carbon footprints and water footprints were analyzed. Additionally, this study also analyzed the gaps in both environmental footprints between current aquatic food consumption and (a) the Dietary Guidelines for Chinese Residents (2022), and (b) EAT-Lancet recommendations. The results aim to reveal the potential environmental benefits of aquatic food consumption patterns in China.

Finally, the results among different provinces were presented via maps. The spatial analyses were performed using the software ArcGIS version 15.1 (Esri China Ltd., Hong Kong, China) for Windows. The provinces of China analyzed in this study included 31 provincial administrative divisions (Chinese provincial administrative divisions includes provinces, municipalities, and autonomous regions). Data for Hong Kong, Macau, and Taiwan are not included due to the unavailability of data.

## 3. Results

### 3.1. Dynamic Characteristics of Aquatic Product Supply and Consumption in China

**Figure 2:**
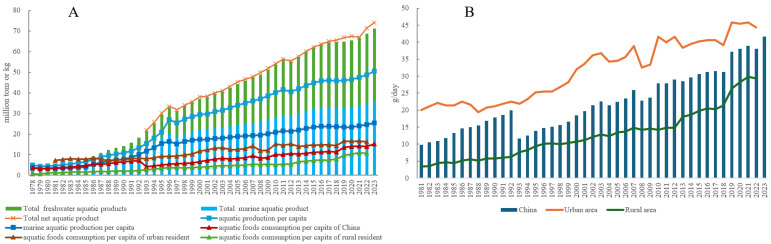
Aquatic food supply and consumption in China. (**A**) Aquatic food supply in China (1978–2023). (**B**) Aquatic food consumption per capita per day for Chinese residents (1981–2023). Data source: Compiled by the author based on data from China Agricultural Statistics 1949–2019, China Fishery Statistics Yearbook (2006–2024), and China Fishery Statistics Bulletin 2023.

Figure 2 illustrates the dynamic characteristics of aquatic product supply and consumption in China. Between 1978 and 2023, the total aquatic product output increased from 4.65 million tons to 71.16 million tons, representing a 14.3-fold increase with an average annual growth rate (AAGR) of 6.25% (Figure 2A). By incorporating imports and exports, the total net aquatic product supply—calculated as imports included and exports excluded—rose from 21.92 million tons in 1993 to 74.13 million tons in 2023, reflecting a 2.38-fold increase with a 4.15% AAGR.

Despite the consistent increase in marine aquatic supply, the proportion of marine aquatic products decreased from 77.25% (3.59 million tons) in 1978 to 50.39% (35.85 million tons) in 2023 (Figure 2A). Similarly, per capita aquatic production increased significantly, while the proportion of marine aquatic products decreased from 76.21% (3.73 kg) in 1978 to 50.42% (25.43 kg) in 2023.

Aquatic food consumption per capita also saw significant growth, rising from 3.50 kg in 1978 to 15.20 kg in 2023. However, a persistent and stable gap remains between rural and urban residents. For instance, in 1981, urban residents consumed 7.30 kg per capita, while rural residents consumed only 1.28 kg. By 2015, rural residents’ average consumption had increased to 7.20 kg per capita. In 2022, the national average per capita aquatic food consumption was 13.9 kg, with rural residents consuming 10.7 kg and urban residents consuming 16.2 kg (Figure 2A).

When converted to daily consumption, the national average in 2022 was 38.08 g/day, while rural residents consumed 29.32 g/day. Both figures are below the Chinese Food Guide Pagoda 2022 recommendation of 42.86–71.43 g/day (300–500 g/week). Urban residents, however, met the recommendation in 2022, averaging 44.38 g/day. Notably, urban residents have consistently met the recommendation since 2019, when their daily consumption reached 45.75 g (Figure 2B).

These dynamic changes in aquatic product supply and consumption indicate that China’s aquatic food supply is adequate to meet consumption needs (Figure 2). Moreover, based on the annual aquatic product consumption per capita (15.2 kg) and population (1.41 billion persons) information, the total aquatic products consumed in 2023 was 21.43 million tons, accounting for 30.10% of the supply (71.16 million tons) in China. Considering the gaps between aquatic production and dietary consumption, the potential for aquatic foods to fulfill the dietary recommendations outlined in the Chinese Food Guide Pagoda 2022 is both promising and sufficient. Further analysis of these gaps is provided in Section 3.2.2.

### 3.2. Aquatic Product Supply Trend and Potential in China

#### 3.2.1. China’s Aquatic Product Supply Potential

**Figure 3:**
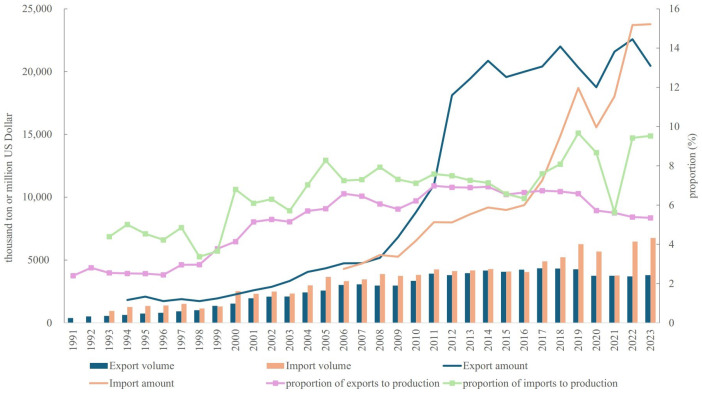
Aquatic products import and export in China (1991–2023). Data source: Compiled by the author based on data from *China Agricultural Statistics 1949–2019*, *China Fishery Statistics Yearbook* (2006–2024), and *China Fishery Statistics Bulletin 2023*.

To assess the supply potential and orientation of aquatic products, this study analyzed trade data and marine fishery production. Figure 3 illustrates the import and export characteristics of aquatic products from 1991 to 2023. Overall, China has consistently maintained a trade deficit in aquatic products (Figure 3). Specifically, the export volume increased from 378.0 thousand tons in 1991 to 3.8 million tons in 2023, representing an 8.05-fold increase with an average annual growth rate (AAGR) of 7.48%. Similarly, import volume rose from 945.0 thousand tons in 1993 to 6.76 million tons in 2023, a 5.16-fold increase with an AAGR of 6.78%.

From a monetary perspective, China has primarily exported high-value aquatic products while importing lower-value ones over the past three decades. This trend shifted slightly after 2022. Between 1994 and 2023, the export trade value increased from $1.82 billion to $20.46 billion, while the import trade value rose from $4.30 billion in 2006 to $23.77 billion in 2023. Interestingly, the proportion of exports to the total trade value remained relatively stable at below 7%, showing an increasing trend from 1991 to 2011 before declining afterward (Figure 3). In contrast, the proportion of imports to total trade value increased from 4.39% to approximately 10%. Although the COVID-19 pandemic disrupted aquatic product trade between 2020 and 2022, the market has since recovered.

**Figure 4:**
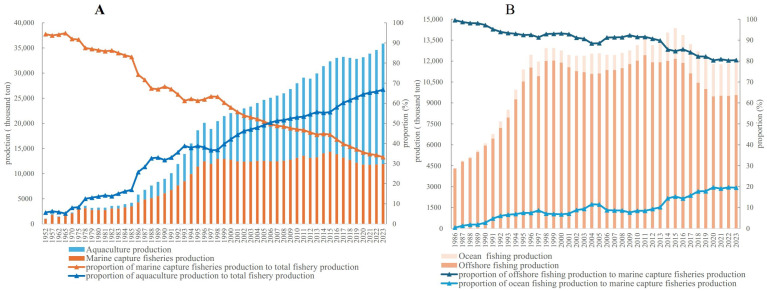
Marine fishery products in China. (**A**) Marine fishery products and its proportion by source in China (1952–2023). (**B**) Marine capture fisheries products and their proportion by source in China (1986–2023). Data source: Compiled by the author based on data from *China Agricultural Statistics 1949–2019*, *China Fishery Statistics Yearbook* (2006–2024), and *China Fishery Statistics Bulletin 2023*.

Despite a decline in the proportion of marine aquatic products (Figure 2A), the total volume of marine fishery production in China has increased (Figure 4A) from 1.06 million tons in 1952 to 35.85 million tons in 2023. However, the data also reveal that aquaculture is becoming the dominant contributor to aquatic product supply. The volume of aquaculture products surpassed marine capture fishery products in 2006. By 2023, aquaculture production reached 23.96 million tons, accounting for 66.82% of marine fishery production, while marine capture fisheries contributed 11.90 million tons.

For marine capture fisheries, the proportion of offshore fishing products has declined, while ocean fishing has shown an upward trend (Figure 4B). Despite this shift, the total volume and proportion of marine capture fishery production have remained stable, with limited growth potential. These findings suggest that aquaculture is poised to become the primary focus of aquatic product supply in China.

#### 3.2.2. Dynamic Changes in the Supply and Consumption of Aquatic Products

**Figure 5:**
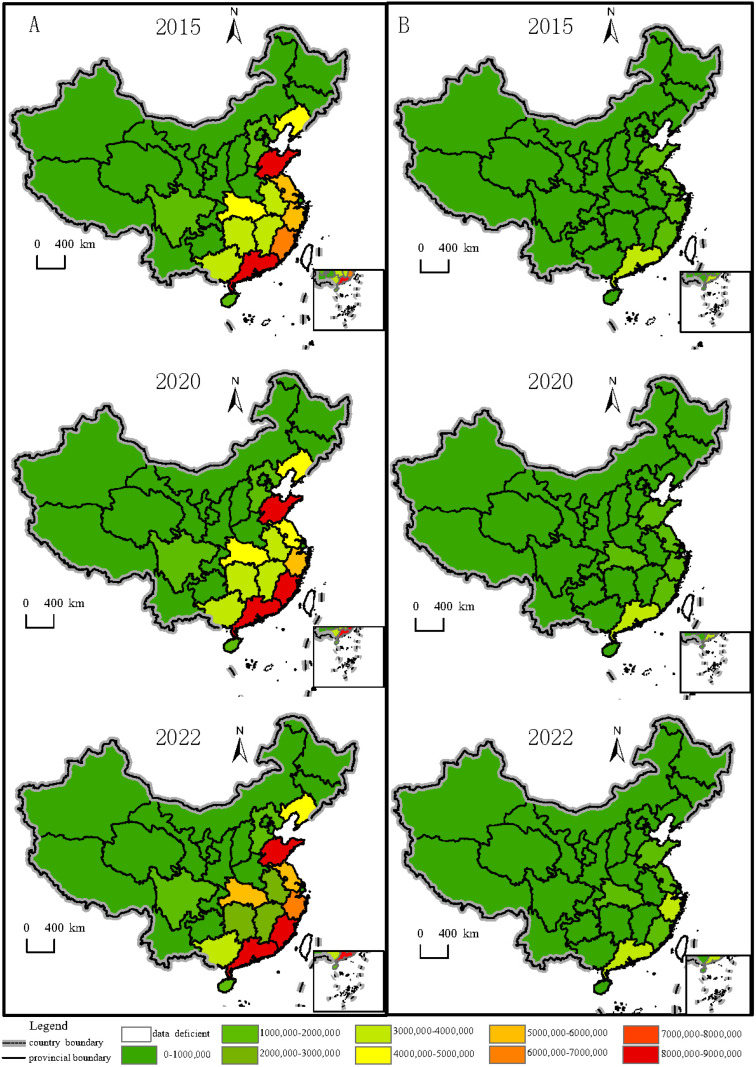
Marine fishery product supply and consumption in China by province. (**A**) Marine fishery product supply in 2015, 2020, and 2022 by province (tons). (**B**) Marine fishery product supply in 2015, 2020, and 2022 by province (tons). Data source: *China Fishery Statistics Yearbook* (2016, 2021, 2023).

In addition to the dynamic changes in the supply and consumption of aquatic products at the national level, similar trends at the provincial level reveal structural patterns in aquatic resource utilization. This study analyzed data on aquatic product supply and consumption by province and showed the temporal and spatial dynamics (Figure 5). The supply of aquatic products in China follows a spatial pattern, with higher levels in coastal provinces and lower levels in inland areas (Figure 5A). Furthermore, the supply of aquatic products is higher in South and East China compared to other regions.

Regarding aquatic product consumption, our results indicate that the current supply structure is sufficient to meet consumption needs (Figure 5B). Coastal provinces such as Guangdong, Zhejiang, Jiangsu, Shandong, Shanghai, and Liaoning tend to consume more. Additionally, Henan Province consumes a larger quantity of aquatic products than other inland provinces, likely due to its large population (9.87 million people in 2022).

Given the high contribution of marine fishery products to China’s total aquatic product supply, the supply of marine products in coastal provinces since 2010 were analyzed (Table 1). The results show that among the 12 coastal provinces, Fujian, Shandong, Zhejiang, Guangdong, and Liaoning contributed the majority of marine product supply, accounting for 80.47% in 2022. Moreover, the AAGR for these provinces remains positive with increasing supply volumes. Notably, Shanghai recorded the highest AAGR of 22.33% for marine fishery supply between 2010 and 2023, growing from 121,464 tons to 1.36 million tons. In contrast, marine fishery products in Beijing and Jiangsu have consistently declined, with Beijing shifting from expansion between 2011 and 2015 to contraction since 2016.

**Figure 6:**
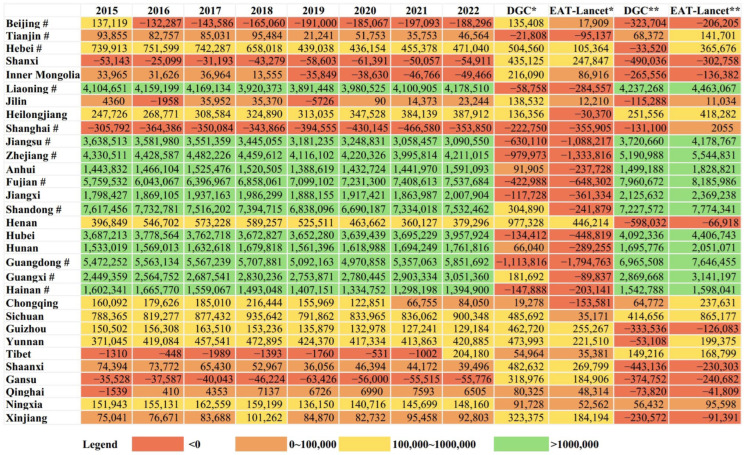
Dynamic surplus of supply and consumption of aquatic products in China by province. Note: unit: tons. **#** means the provinces are coastal provinces. * means the columns are the values of gaps between adjusted aquatic product consumption amount (according to DGC or EAT-Lancet) and the current aquatic product consumption by province in 2022. ** means the columns are the values of the gaps between adjusted aquatic product consumption amount (according to DGC or EAT-Lancet) and the current aquatic product supply by province in 2022. Abbreviation: DGC—Dietary Guidelines for Chinese Residents (2022).

This study also analyzed the gaps between aquatic product consumption and supply for each province (Figure 6). Provinces such as Shaanxi, Shanghai, and Gansu have consistently faced deficits in aquatic product supply. Beijing has been in deficit since 2016, and Inner Mongolia joined the deficit group in 2019. However, Tibet reversed its deficit in 2022, after experiencing shortages since 2015.

When adjusted for the Dietary Guidelines for Chinese Residents (2022) and the EAT-Lancet recommendations, deficits remain in Beijing, Shanghai, Shanxi, Inner Mongolia, and Gansu. Additionally, provinces such as Hebei, Liaoning, Guizhou, Yunnan, Shaanxi, Qinghai, and Xinjiang also face supply deficits (see the last two columns in Figure 6).

### 3.3. Environmental Sustainability of Aquatic Food Consumption

**Figure 7:**
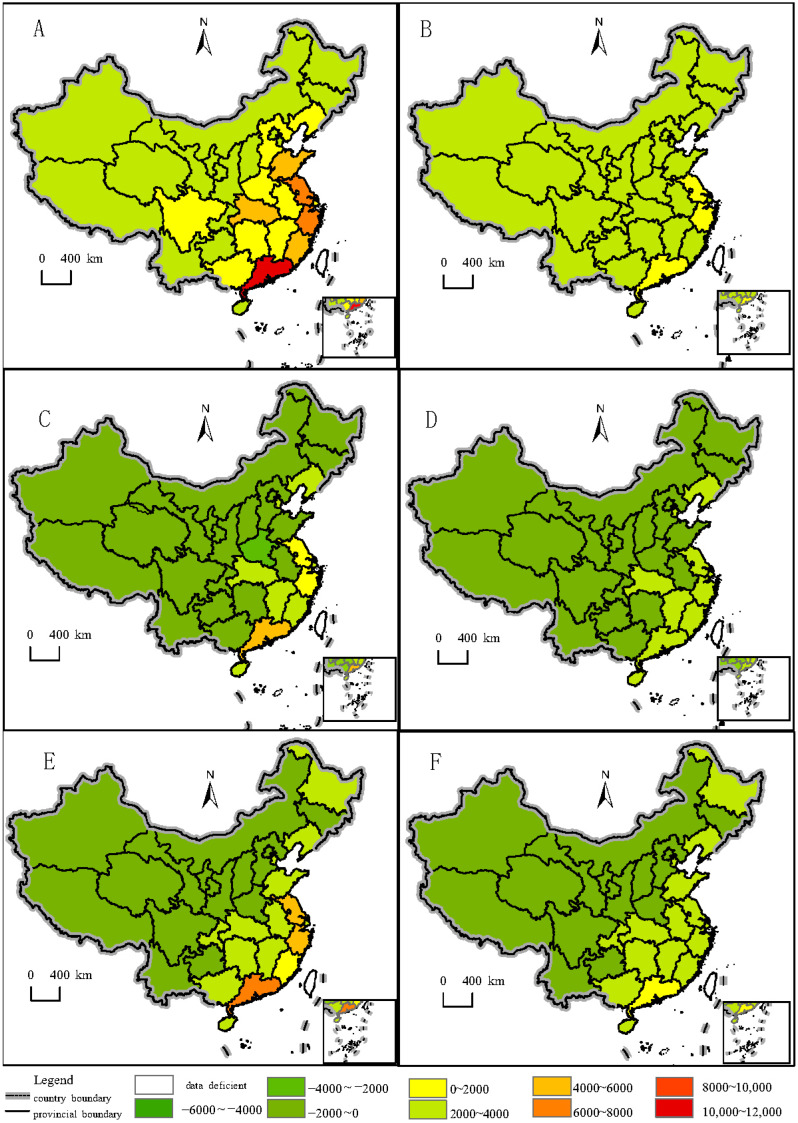
Carbon footprints and water footprints of Chinese residents’ aquatic product consumption by province in 2022. (**A**) Carbon footprint of aquatic product consumption by province (2022). (**B**) Water footprint of aquatic product consumption by province (2022). (**C**) Carbon footprint of gaps between adjusted aquatic product consumption amount according to DGC and the current aquatic product consumption by province in 2022. (**D**) Water footprint of gaps between adjusted aquatic product consumption amount according to DGC and the current aquatic product consumption by province in 2022. (**E**) Carbon footprint of gaps between adjusted aquatic product consumption amount according to EAT-Lancet and the current aquatic product consumption by province in 2022. (**F**) Water footprint of gaps between adjusted aquatic product consumption amount according to EAT-Lancet and the current aquatic product consumption by province in 2022.

To assess the environmental sustainability of current aquatic food consumption patterns in China, carbon and water footprints were analyzed. In 2022, the total carbon footprint of aquatic food consumption was 76,463.78 kg CO_2_-eq, and the water footprint was 24,230.08 m^3^. An analysis of provincial carbon footprints revealed that coastal provinces such as Guangdong, Jiangsu, Zhejiang, Shanghai, Fujian, and Shandong, along with Henan (a densely populated inland province), had relatively high carbon footprints (Figure 7A). Similarly, Guangdong, Zhejiang, and Jiangsu were identified as provinces with high water footprints from aquatic food consumption (Figure 7B).

To evaluate potential improvements, per capita aquatic product consumption in each province was adjusted based on the Dietary Guidelines for Chinese Residents (2022) and the EAT-Lancet recommendations, considering the population of each province in 2022. Under these new consumption scenarios, the distribution of carbon and water footprints was recalculated. The consumption gaps shown in Figure 6 suggest two opposing outcomes.

When adjusted to meet the Dietary Guidelines for Chinese Residents (2022) recommendations (at least 300 g per week per capita), total aquatic food consumption would increase by 2,131,378 tons, along with a rise in the carbon footprint of 8205.81 kg CO_2_-eq and a water footprint increase of 2600.28 m^3^. On the other hand, when adjusted to align with the EAT-Lancet recommendations (28 g per day per capita), total aquatic food consumption would decrease by 5,453,077 tons, while the carbon footprint would increase by 20,994.34 kg CO_2_-eq, and the water footprint would rise by 6652.75 m^3^.

Comparisons between the adjusted and current consumption patterns reveal the gaps, as shown in Figure 7C,D. Figure 7C illustrates the differences in carbon footprints between the adjusted and current patterns by province, while Figure 7D shows the corresponding gaps in water footprints. The results indicate that in this scenario, only Guangdong, Jiangsu, Zhejiang, Shandong, Shanghai, Tianjin, Fujian, Jiangxi, and Hubei experienced significant reductions in both carbon and water footprints.

Furthermore, similar adjustments were made to align with the EAT-Lancet recommendations, and the resulting gaps are shown in Figure 7E,F. In this scenario, more provinces showed reductions in both carbon and water footprints, including Heilongjiang, Liaoning, Shandong, Tianjin, Shanghai, Zhejiang, Anhui, Fujian, Jiangxi, Hubei, Hunan, Guangxi, Guangdong, Hainan, and Chongqing.

## 4. Discussions

### 4.1. The Role of Aquatic Products in Food System Transformation in China and Other Countries

There is a coupling relationship between residents’ food consumption, agriculture, and the ecosystem [41,42,43,44]. This same relationship applies to aquatic food. The consumption patterns of aquatic products are influenced by fishery supply, and, in turn, the supply and consumption patterns of aquatic products impact the marine ecosystem [27,45]. Our research, based on China’s aquatic products, explores the consumption patterns of aquatic foods, their temporal and spatial variations, and the environmental effects they cause. Through these analyses, this study aims to provide valuable insights for the transformation of China’s food system. The findings of this study are consistent with previous studies that have highlighted the growing importance of aquaculture as a more sustainable alternative to traditional marine fisheries [46], which have faced challenges such as overfishing and habitat degradation [47]. However, this study adds value by examining both the environmental impacts and the dietary implications of consumption patterns in a comprehensive, multi-decade context.

Additionally, this study underscores the importance of addressing regional imbalances in supply and consumption. While the overall supply of aquatic products is sufficient to meet national needs, disparities remain, particularly in inland areas where access to aquatic foods is limited [48]. This highlights the need for targeted policy interventions and structural adjustments to ensure that all regions benefit from sustainable aquatic food systems. Previous research has often focused on the national or global scale [2,46,49], but this study’s focus on provincial-level disparities adds a layer of specificity that could inform more effective local-level strategies.

### 4.2. Implications of This Study

As the world’s largest producer, trader, and consumer of fishery products, China’s current fishery development model is unsustainable [50,51]. Overfishing is widespread in the fishery sector, and maintaining the current high yields through traditional marine fishing is environmentally unsustainable [49,52,53]. This model has led to a series of ecological and environmental issues, such as the decline in fish populations, loss of marine biodiversity, and disruption of ecosystem balance [12]. From a dietary need perspective, this study found that China’s current fishery production is already sufficient to meet the country’s food security requirements. The current level of fishery exploitation is adequate, and there is no need to increase it further. What is needed now is effective structural adjustment, market development, and more environmentally friendly practices, such as sustainable aquaculture, to address the ecological and environmental challenges caused by overfishing. This study provides evidence from China to explore the potential of aquatic products for food system transformation. Further expansion of marine fisheries could have negative impacts on marine ecological security in the future. Many countries, such as Norway, Japan, and Korea, are aware of these problems [54]. To address the problem of overfishing, many countries have already introduced relevant laws and regulations to limit its occurrence [1,35].

In addition, this study also highlights the potential of aquatic products in transforming China’s food system. Previous research has often focused separately on either the environmental impacts of aquatic food production or dietary shifts [55], but this study integrates both aspects, offering a more holistic view of how sustainable aquatic product management can contribute to food system transformation. The current supply of aquatic products is sufficient to meet food consumption needs. Previous research has shown that farmed fish has only 1/12 the carbon footprint of beef [56]. In the future, emissions can be reduced by adjusting dietary patterns—specifically by increasing aquatic product consumption and reducing beef intake, without compromising nutritional requirements—leading to positive ecological outcomes. Given the current situation in China, this approach is both feasible and holds significant potential. The findings of this study offer valuable insights for the development of food system transformation strategies, as well as carbon and emission reduction policies, not only in China but also for other countries and global efforts in the future.

Moreover, this research is particularly timely, as global concerns about climate change, overfishing, and resource depletion continue to grow [57,58,59,60,61]. This study offers concrete evidence of how sustainable consumption and production practices in China’s aquatic food sector could contribute to broader global goals, such as achieving the United Nations Sustainable Development Goals (SDGs) related to responsible consumption and production (SDG 12), climate action (SDG 13), and life below water (SDG 14) [62].

Last but not least, based on the research findings of this study, to achieve sustainable dietary and environmental goals, several policy recommendations can be raised. (1) Promote dietary shifts toward sustainable aquatic products. For example, encourage consumers to replace high-carbon foods (e.g., beef) with sustainable aquatic products through public awareness campaigns and subsidies. (2) Prioritize the development of aquaculture. The results from this study revealed that aquaculture could be one of the potential approaches to address regional supply deficits and reduce reliance on unsustainable marine fisheries. Investing in offshore aquaculture technologies and infrastructure, particularly in inland regions with supply deficits, could be a promising strategy. (3) Pay attention to regional equity when formulating development policies. Develop targeted policies to address uneven supply and demand distribution, such as improving transportation networks for aquatic products. These actions will not only enhance food security but also contribute to global efforts to combat climate change and resource depletion.

### 4.3. Limitations of This Study

The author acknowledges several limitations in this study. Although this study primarily relies on statistical data to analyze the characteristics of China’s aquatic product supply and consumption structure, as well as its resource and environmental impacts, a causal analysis of the factors driving the dynamic changes in China’s aquatic product supply and consumption patterns was unable to conduct due to limitations in data availability. The author hopes that, as the team’s research progresses and access to data improves, it will be possible to expand on this topic in the future.

Additionally, this study exclusively uses statistical data and information from open-access platforms, which involve different adjustment methods. These variations may affect the accuracy and reliability of the results. Since the statistical yearbook data derived from household sample surveys were taken nationwide by survey teams of the statistical bureaus of every county, the author believes the results of the data represent the real-life conditions of Chinese residents. However, despite these limitations, our study provides a comprehensive overview of the dynamic changes in aquatic products in China over the past few decades.

### 4.4. Potential Directions for Future Research

Future research should focus on addressing the limitations identified in this study, particularly in terms of refining data collection and exploring causal relationships between consumption behaviors and environmental impacts. Additionally, investigating the long-term ecological effects of different aquaculture methods and exploring the potential for policy interventions that incentivize more sustainable production and consumption practices would further support effective policy development. Furthermore, integrating social, economic, and cultural dimensions into the analysis of sustainable consumption will provide a more holistic view of how to achieve food system transformation.

## 5. Conclusions

This study analyzes the supply, consumption, and environmental sustainability of aquatic products in China. Between 1952 and 2023, aquatic product output increased significantly, but disparities remain between rural and urban consumption patterns. While China’s aquatic food supply is sufficient to meet national needs, the distribution of supply and demand remains uneven, particularly in inland regions. The environmental analysis revealed that current consumption patterns have high carbon footprints and water footprints. However, adjusting consumption based on the Dietary Guidelines for Chinese Residents (2022) and EAT-Lancet recommendations could reduce environmental impacts. Shifting from high-carbon foods like beef to more sustainable aquatic products could improve both environmental outcomes and dietary health.

This study also highlighted regional deficits in aquatic product supply, suggesting the need for targeted strategies, such as promoting aquaculture, dietary shifts, and regional equity. These changes are critical for balancing food security, health, and ecological sustainability in China’s food system. This study provides valuable insights for both policymakers and researchers aiming to promote sustainable food systems. By linking dietary health and environmental sustainability, the findings suggest actionable steps for transforming China’s aquatic food sector. However, the study’s reliance on available data limits the granularity of regional consumption patterns, and further research is needed to address socio-economic and cultural factors influencing consumption behaviors. Additionally, challenges related to scaling up sustainable aquaculture practices require further investigation. Future research should refine data collection and explore causal relationships to support more effective policy development. Nonetheless, this study demonstrates the potential for transforming China’s aquatic food system to achieve sustainable dietary and environmental goals.

## Figures and Tables

**Table 1:** Marine product supply in China’s coastal provinces (2010–2022).

Year	Beijing	Tianjin	Hebei	Liaoning	Shanghai	Jiangsu	Zhejiang	Fujian	Shandong	Guangdong	Guangxi	Hainan
2010	8960	38,986	582,600	3,497,366	121,464	1,364,468	3,812,332	5,127,982	6,463,345	4,015,032	1,544,481	1,178,877
2011	7532	38,342	563,281	3,657,958	121,586	1,420,840	4,109,846	5,261,619	6,647,212	4,182,257	1,593,225	1,240,420
2012	212,580	38,393	634,631	3,469,492	132,103	1,484,183	4,131,504	5,189,852	6,524,046	4,102,922	1,622,943	1,353,555
2013	232,023	68,377	682,809	3,642,478	134,782	1,505,920	4,249,060	5,434,711	6,654,179	4,195,353	1,683,926	1,439,888
2014	274,112	68,394	731,594	3,858,458	178,756	1,504,730	4,487,041	5,738,022	7,085,761	4,271,819	1,717,947	1,445,941
2015	298,020	66,510	760,931	3,833,843	169,530	1,481,856	4,676,922	6,053,890	7,352,063	4,351,448	1,769,963	1,507,990
2016	13,514	60,954	806,799	3,923,995	141,833	1,472,415	4,700,757	6,332,421	7,541,952	4,415,356	1,844,908	1,560,284
2017	9000	48,589	811,407	3,918,774	144,701	1,487,281	4,723,721	6,624,580	7,371,727	4,518,133	1,919,010	1,448,853
2018	1706	48,695	767,665	3,670,133	166,632	1,408,306	4,632,465	6,968,161	7,360,685	4,491,690	1,944,161	1,378,071
2019	6661	40,080	695,640	3,699,340	195,729	1,370,205	4,436,164	7,235,283	7,062,086	4,554,912	1,994,915	1,350,103
2020	5548	42,784	659,744	3,778,492	160,732	1,349,896	4,509,396	7,404,915	7,180,937	4,505,267	2,009,180	1,281,161
2021	7472	43,331	734,054	3,970,297	159,424	1,306,286	4,570,248	7,574,863	7,403,008	4,550,424	2,085,282	1,273,762
2022	4000	41,000	770,600	4,027,000	1,364,000	1,352,000	4,754,000	7,633,929	7,622,454	4,583,000	2,132,869	1,280,529
AAGR	−6.50	0.42	2.36	1.18	22.33	−0.08	1.86	3.37	1.38	1.11	2.73	0.69

Unit: tons; Data source: *China Fishery Statistics Yearbook* (2011–2023); Abbreviation: AAGR—average annual growth rate.

## Data Availability

The original contributions presented in this study are included in the article. Further inquiries can be directed to the corresponding author.
